# Supplementary material for: Splice-Junction-Based Mapping of Alternative Isoforms in the Human Proteome
Source: Cell Rep. Author manuscript; Available in PMC 2020 Jan 15. (PMC6961840; doi:10.1016/j.celrep.2019.11.026)
Supplement: 2 [file NIHMS1546469-supplement-2.zip › DF1/PXD010154/Liver/Liver_22_DOK2_LGPAGAAGGPGEASSVFLYLQQQQTFGK.pdf]

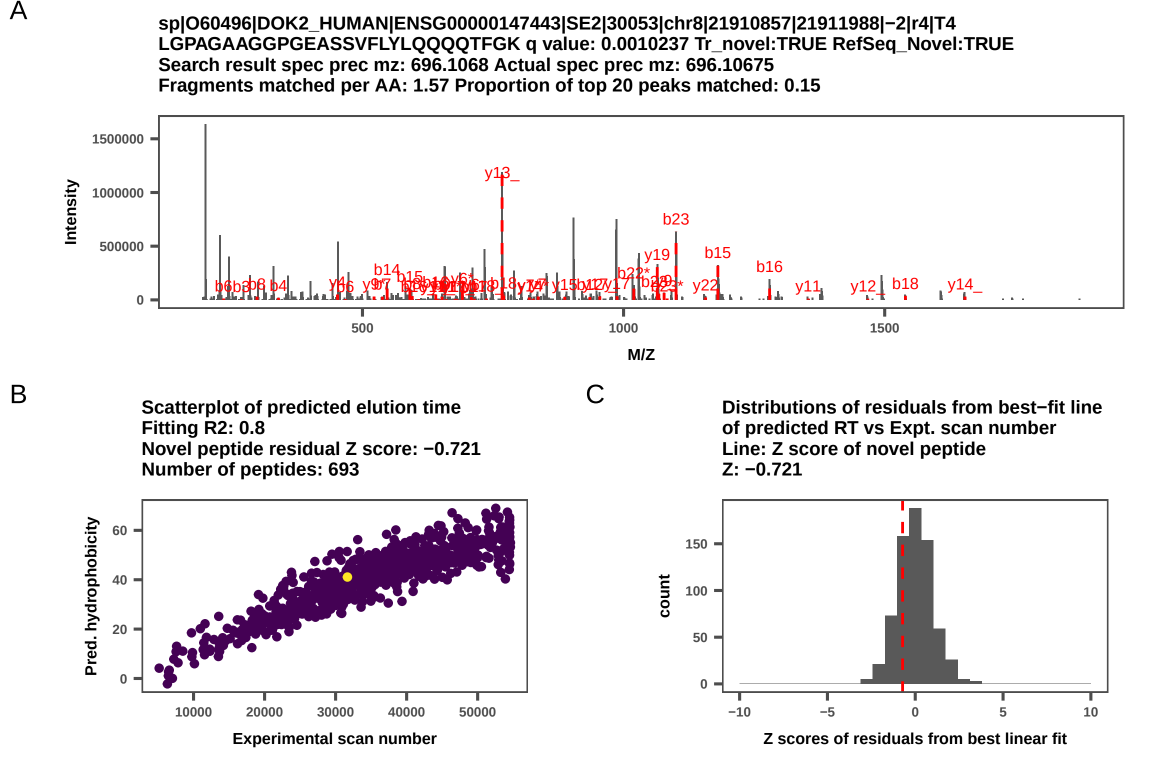
A
sp|O60496|DOK2_HUMAN|ENSG00000147443|SE2|30053|chr8|21910857|21911988|−2|r4|T4
LGPAGAAGGPGEASSVFLYLQQQTFGK q value: 0.0010237 Tr_novel:TRUE RefSeq_Novel:TRUE
Search result spec prec mz: 696.1068 Actual spec prec mz: 696.10675
Fragments matched per AA: 1.57 Proportion of top 20 peaks matched: 0.15
Intensity
1500000
1000000
500000
0
M/Z
500
1000
1500
y13_
b23
y19
b15
b16
b14
b15
y11
y12_
b18
y14_
y22
b22*
B
Scatterplot of predicted elution time
Fitting R2: 0.8
Novel peptide residual Z score: −0.721
Number of peptides: 693
Pred. hydrophobicity
60
40
20
0
10000
20000
30000
40000
50000
Experimental scan number
C
Distributions of residuals from best−fit line
of predicted RT vs Expt. scan number
Line: Z score of novel peptide
Z: −0.721
count
150
100
50
0
−10
−5
0
5
10
Z scores of residuals from best linear fit
